# Non-pharmaceutical Interventions and Social Distancing as Intersubjective Care and Collective Protection

**DOI:** 10.1007/s41649-022-00212-7

**Published:** 2022-08-13

**Authors:** Corrado Piroddi

**Affiliations:** Faculty of Social Science, Tampere University, Tampere, Finland

**Keywords:** Care, Honneth, Recognition, Social distancing, Vulnerability

## Abstract

The paper discusses non-pharmaceutical interventions (NPIs) as a collective form of protection that, in terms of health justice, benefits groups at risk, allowing them to engage in social life and activities during health crises. More specifically, the paper asserts that NPIs that realize social distancing are justifiable insofar as they are constitutive of a type of social protection that allows everyone, especially social disadvantaged agents, to access the public health sphere and other fundamental social spheres, such as the family and civil society.

In the last years, which have been characterized by the SARS-CoV-2 pandemic, many ethical theories have discussed problems related to the implementation of non-pharmaceutical interventions (NPIs) in terms of restrictions on negative freedom, individual and civil rights, and self-determination. The present paper aims to provide a viewpoint that differs from those perspectives, which frame many public health strategies as ethically challenging, due to the infringement on individual freedom and the limitations on civil rights that they provoke. In doing so, the paper will employ Axel Honneth’s ethical perspective, which is mainly focused on the normative idea of individual self-flourishing and the collective realization of social freedom.

The main hypothesis of this work is that the specific features of Honneth’s thought make it interesting to ethical and bioethical reflections concerning the moral status of NPIs and social distancing. The paradigm of recognition emphasizes the analysis of the duties and rights that we have in terms of our interpersonal relations, which are not necessarily legitimized by public authorities or valuable because of their legal value. In addition, it enlarges and deepens the idea of freedom in a way that exceeds the traditional conceptions of freedom as negative liberty and freedom in relation to self-determination. Introducing the idea of social freedom and enabling duties, Honneth’s perspective could allow us to describe and evaluate social distancing and NPIs in terms of interpersonal and collective protection, instead of demoting them to arbitrary forms of limitations and the negation of freedom.

In this regard, the paper aspires to provide ethical considerations that, despite stemming from a philosophical tradition that differs from the liberal and contractualist one, are consistent with numerous recent works by many non-consequentialist theorists of freedom. For instance, Oberman (2022) argues that NPIs and lockdowns can be justified precisely because they “could increase overall freedom, protect more valuable freedoms, or improve the distribution of freedom.” Others (John and Curran 2021; Kugelberg 2021) maintain that such health measures are morally justifiable because the cost–benefit analysis, personal value choice, and individual freedom are outweighed by other interpersonal and ethical considerations.

In line with these ideas, the paper will discuss NPIs as a collective form of protection that, in terms of health justice, benefits at-risk groups, allowing them to engage in social life and activities during health crises. More specifically, the paper will assert that NPIs that realize social distancing are justifiable insofar as they are constitutive of a type of social protection that allows everyone, especially social disadvantaged agents, to access the public health sphere and other fundamental social spheres when these same social agents are threatened by outbreaks, epidemics, and pandemics that are unexpected or difficult to curb. It will be argued that social distancing is not only constitutive of a very peculiar form of social cooperation that aims at preserving and protecting the health of the population. Social distancing also reflects a specific form of interpersonal recognition: care toward others’ vulnerability. Such a form of recognition is regulated by the idea that I owe a duty to safeguard everyone’s else health in a situation in which the spread of a pathogenic agent cannot be managed through therapies, vaccination campaigns, and track-and-trace systems, affecting a society that is already characterized by many profound health inequalities.

First, the paper will introduce some conceptual distinctions concerning NPIs, lockdown, and measures of control, briefly explaining their public health ratio. In the second section, the paper will describe Honneth’s theory of recognition and concept of social freedom. The third section attempts to explain how, through the ideas of recognition and social freedom, NPIs and social distancing can be seen and justified as social practices that aim to protect our neighbors in circumstances of health crisis and a lack of preparedness. The following pages will attempt to depict them as intersubjective protections that mitigate health inequalities while guaranteeing participation in basic social life for vulnerable subjects.

## What NPIs are: Definition and Aims

Before discussing the moral questions related to the implementation of NPIs from Honneth’s perspective, it is important to clarify the differences between the measures of protection against SARS-CoV-2, the measures aimed at controlling the implementation of the former, and the so-called lockdown measures. Non-pharmaceutical interventions constitute a set of measures aimed at curbing virus circulation and epidemic spread: physical distancing, staying at home if sick, quarantine, isolation, the avoidance of crowded places, mask usage, and travel restrictions. At the beginning of the pandemic, there was uncertainty regarding whether NPIs would be effective. Now, we know that different types of NPIs have different impacts on SARS-CoV-2 community transmission. Some of them, such as travel restrictions, are more effective than others, for example, the cleaning and disinfection of shared surfaces (Haug et al. 2020; Brauner et al. 2021; Perra 2021). Each intervention has some imperfections. Their contemporary implementation, however, could increase the chances of success in reducing the impact of highly infective pathogens, without fully guaranteeing complete effectiveness. In addition, it is worth noting that their implementation requires the active participation of citizens. State power and collective agents, such as companies, labor unions, and many private and public institutions, can either recommend these interventions or impose them through coercion. However, this top-down agency is not sufficient. The efficient implementation of NPIs requires citizens’ acknowledgment: they should be akin to accepting temporary restrictions on their civil and political freedoms in the name of the common good and social solidarity.

The measures of control and surveillance regarding the infections include immunity passports, vaccine passports, and test-and-trace procedures. The implementation of these measures raises issues that concern not only restrictions on freedom but also privacy protection. They will not be discussed in the following pages, which will be focused on social distancing and lockdown as forms of collective and interpersonal protection.

A lockdown (LD) can be conceived of as a stay-at-home order directed at the entire population of an area, often combined with non-essential business closures, school closures, and a round-the-clock curfew. Lockdowns should not be conceived as something desirable or the silver bullet that can put an end to a pandemic of such a scale. The lockdowns are a *stage* of a potential containment strategy, not its ending point. Furthermore, they can be seen as a sign of defeat, a drastic public health policy solution that signals the failure of preparedness plans. Governments enact lockdowns as an extreme measure, for example, when the hospital system is at risk of collapse. Such a situation indicates that not enough has been done previously to protect healthcare structures or there were flaws and inaccuracies in the risk assessment. Are the LDs alone enough? No, because reopening requires the implementation of strategies of mitigation, eradication, or elimination (test-and-trace; a vaccination campaign; securing schools, public places, and workplaces) that ensure the circulation of the pathogen is low or absent. A lockdown helps to gain a few weeks of hospital autonomy. Without any wider elimination or mitigation strategy, its beneficial effects are nullified in a short time.

Generally, the implementation of NPIs and lockdowns has three objectives. The first is related to the biophysical evolution of SARS-CoV-2 (Lobinska et al. 2022). After almost 2 years, we know that every infection represents a chance of mutation and evolution on the part of the virus. In this respect, not limiting the virus’s circulation can constitute the backdrop for the emergence of virus strains that are more transmissible or capable of bypassing immunization. The implementation of NPIs is thus important for limiting the opportunity of the mutation of the virus, which we do not understand well enough from a biological and evolutionary perspective. It is not the case that the variants that have been dominant until now emerged in contexts in which there were no or poor restrictions and virus circulation was high.

The second objective is preventing excess load on the public healthcare system by reducing viral circulation. SARS-CoV-2 treatment often requires hospitalization. According to clinical guidance by WHO (2021: 9–10), about 15% who are infected with SARS-CoV-2 become seriously ill and require supplemental oxygen. Five percent of sick people become critically ill and require intensive care. In this regard, NPIs are fundamental to keeping virus circulation under control and, consequently, maintaining public health structures’ functionality. This is not a question of choosing between social distancing or, for instance, cancer screening. Quite to the contrary, society needs NPIs and, in extreme cases, lockdowns in order to treat cancer when a pathogen threatens the overall stability of society. When COVID-19 cases rose in our hospitals, this left fewer resources for cancer patients, and ending chemotherapy entirely was discussed as a possibility. A society in which the healthcare system does not work is a society that is at risk of collapsing.

Finally, NPIs are meant to protect everyone’s health, especially the most vulnerable individuals, social groups, and classes. Now, it is widely accepted by that SARS-CoV-2 is especially dangerous to subjects who belong to groups at risk. Elderly individuals and people who suffer from pre-existing, underlying conditions (heart disease, diabetes, lung diseases, leukemia, liver disease, and obesity) are highly predisposed to severe COVID-19. Nevertheless, social marginalization and poor socio-economic conditions should be included among the risk factors as well. It has been shown that there are health inequities concerning increased morbidity and mortality from respiratory-transmitted infectious diseases because of social factors (Bansal et al. 2021). Particularly for flu, it is now evident that variations in social and healthcare determinants sharpen epidemics of this seasonal disease. Inequities and low socio-economic status are tendentially associated with the higher transmission of flu. The disproportionate disease burden in the poorest sectors of the population is driven by factors such as social contact differences, low vaccine uptake, higher susceptibility related to the nature of the work environment, low healthcare utilization, and low sickness absenteeism (Bansal et al. 2021: 12). Such an epidemiological situation is likely to be analogous to the case of COVID-19, whose respiratory pathogenicity, transmissibility, and mortality rate are estimated to be far higher than those related to most strains of the flu (Stojanovic et al. 2021; Piroth et al. 2021). There is already evidence that, in the USA, COVID-19 has a disproportionate impact on non-White populations, which are often victims of structural inequalities in terms of access to medical insurance, a stable income, and wealth (Raifman and Raifman 2020; Acosta et al. 2021).

With these distinctions and aims in mind, we can attempt to understand whether the implementation of social distancing measures can be justified from an ethical perspective that gives priority to the realization of the preconditions for an individual good life and collective cooperation. In this respect, it is useful to introduce Axel Honneth’s paradigm of recognition. Such a perspective considers the realization of good relationships, in terms of intersubjective and social recognition, as being fundamental to human agency and well-being. Considering how much social distancing and NPIs affect our social interactions, Axel Honneth’s ethics can be a fruitful framework for discussing the pros and cons of public health policies based on the actuation of such measures.

## Honneth’s Theory of Recognition: Intersubjectivity and Social Freedom

According to Axel Honneth, both individual self-realization and social cooperation are possible thanks to the role-taking capacity of human beings. Our ability to perceive others as our proper partners of interaction and develop beliefs regarding their empirical and normative expectations (What are my partners in interaction expecting me to do in these circumstances? What do they think about what I should to do in these circumstances?) allows us to enact forms of social relations that constitute the preconditions for both self-realization and collective cooperation (Honneth 2011: 402).

From the point of view of individual self-realization, to enact good and proper forms of recognition means perceiving somebody as a subject who deserves love for their emotional needs, esteem for their contribution to social wealth, respect for their capacity for self-determination and rational decision, and to act consequently (Honneth 1994: 92–93). An act of love is a disinterested one, through which we want to work in favor of the good life and the psychological well-being of beloved persons, without looking for any reward in return. Likewise, to tribute of respect to other persons means interacting with them respect for their capacity to act autonomously and some basic legal rights that protect their individual dignity. Recognizing others properly means empowering them and realizing the preconditions that support individual flourishing (Honneth 2000: 516).

For Honneth, relationships of recognition based on love, esteem, and respect are necessary for developing self-confidence, self-esteem, and self-respect and, therefore, a positive image of the self and its worth. Without loving relationships, it is difficult for one to believe that they can love and take care of themself properly. My self-esteem for my professional achievements and contribution to the well-being of society is fed by the proof of appreciation that I receive from people around me.

Similarly, Honneth considers the same relationships of recognition to be pivotal for the realization of complex forms of social cooperation and social freedom, which, in Hegelian jargon, could be defined as “being-with-oneself-in-the-other.” The delineation of the idea of social freedom is related to Honneth’s attempt to sketch a socio-historical analysis of the origin and development of the idea of individual freedom. The latter can be considered one of the main cornerstones of modern societies. It is not the case that individual freedom is often evoked in denouncing the downsides of the implementation of NPIs. However, it is unclear what the preconditions for individual freedom are. For Honneth (2014), social freedom is that form of liberty that is pivotal in the realization of integral human freedom. Honneth identifies two other potential preconditions in addition to social freedom: negative freedom and reflexive freedom. The term “negative freedom” refers to those negative and legal rights that create a negative duty for socio-political institutions, collective agents, and individuals not to interfere with the personal choices and plans of others. Conversely, the same set of legal rights allows individuals to realize courses of actions that express their preferences and particular interests, despite the influences of their social duties and burdens (Honneth 2014: 70–94). With the notion of “reflexive freedom,” Honneth refers to our capacity to be autonomous moral agents that are able to normatively justify their practical choices in social contexts that are not organized and ruled by positive laws (Honneth 2014: 95–120). Honneth considers negative and reflexive freedom to be necessary for realizing individual freedom in modern and contemporary societies. However, such forms of freedom are also partial and one-sided. They allow individuals to either retreat into their private spheres and realize their interests or reflexively criticize the normative legitimacy of social practices and institutions in which they are involved in their everyday lives. They are partial because individual freedom cannot be realized, regardless of the social and cooperative practices that individuals are either escaping from through negative freedom or criticizing through the exercise of reflexive freedom. For Honneth, social freedom is the basic precondition for realizing individual freedom, but what exactly is social freedom?

Following Honneth (2014: 131–335), social freedom is that kind of freedom that is realized by human beings through those forms of cooperation that are constitutive of the spheres of personal relationships, the market economy, and democratic will-formation. These spheres permit the realization of freedom in a two-sided manner. Acting in them, individuals can contribute to the reproduction of society. At the same time, in these social spheres, they can pursue individual ends that would not be possible to realize without the support of others. In this respect, the realization of social freedom implies the co-presence of the two following aspects. On one hand, individuals should perceive themselves as free agents when realizing forms of cooperation. Social freedom is therefore realized when agents perceive and understand their quest for individual goals as not being limited by but, rather, depending on others also freely pursuing their individual goals. On the other hand, the realization of social freedom entails that everyone can concretely realize their own ends in an integrated society, in which everyone can perceive the fulfillment of their ends as meaningful and necessary for the realization of the ends of all others.

This last requirement is especially important. It presupposes the collective acceptation of intersubjective shared duties and responsibilities by the members of a given society. More specifically, it implies that agents understand that the forms of cooperation enacted in these social spheres rely on duties that are constitutive of the spheres themselves. The duties at work here are thus enabling or liberating. As Joseph Raz states, they permit the existence of valuable relationships and collective activities that create opportunities and options for us, enriching our individual existences. The activities that constitute the spheres of the family, the labor market, and democratic participation are essentially characterized by the duties that they imply. For instance, friendships and love relationships imply duties of reciprocity, aid, and support that go beyond “the general duty to help people in need” (Raz 1989: 19).

Honneth (2014: 123–129) underscores how the opportunity to realize negative freedom, reflexive freedom, and social freedom always requires reciprocal recognition between agents. In all these cases, freedom can be realized only if, on the basis of shared norms and principles, subjects attribute to one another a normative status based on reciprocal regard. In the case of negative freedom, agents can benefit from an egocentric sphere of action, in which they can realize their personal and strategic interests despite other social burdens and duties, insomuch as they respect one another as juridical persons. This form of recognition pushes us to respect the choices of others even though they conflict with our ethical beliefs, to the extent that such practical choices do not break rules. Analogously, reflexive freedom presupposes that, in social situations that are not ruled by positive rights, subjects ascribe to one another the status of moral autonomy and rational self-legislation. According to this status, subjects can act and justify, reciprocally and intersubjectively, their decisions according to moral norms and beliefs that they consider fair and universalizable. Reflexive freedom allows social subjects to justify their practical conduct in the absence of clear negative duties and positive legislation, as well as to criticize the moral legitimacy of existing social practices in the name of their self-scrutiny capacity.

In the case of social freedom, reciprocal recognition seems to imply something more demanding in terms of empirical and normative expectations. In the spheres of social freedom, an agent *x* expects, from their partners of interaction *(y, z…n)*, the realization of behavior that supports *x* in achieving *x*’s ends. In the spheres of intimate relationships, the market, and democratic will formation, I do not expect simply others to respect my strategic and practical choices in the name of my negative rights or self-determination. I expect that they actively support me in the realization of my ends by means of their behavior. For instance, in the private sphere of the family, I do not merely expect my relatives and friends to tolerate my emotional choices, sexual preferences, and gender identity. I expect them to give me all the emotional support I deserve in order to completely and actively realize all these aspects of myself. In the same manner, in the sphere of economic production, I expect others to respect my professional activity, even if teaching and performing research is not profitable from the commodities market’s perspective. If I have such empirical and normative expectations toward others, it is clear that the latter expect that I behave so as to support them in their efforts to realize their personal ends and projects. The duties I owe once I am involved in the social practices that constitute the private sphere, civil society, and the democratic sphere of political participation are ethically compelling insofar as, without them, reciprocal support and cooperation in action there would not be possible.

The point that Honneth (2014) aims to clarify is that both negative and reflexive liberties are always exercised according to the limits imposed by social practices, norms, and institutional facts that individuals cannot conceive as outcomes of their rational choices or subjective desires. Negative freedom can be exercised against the norms and practices that constitute social freedom, implying that the private sphere can bracket the duties related to family cooperation, the division of social labor, participation in the sphere of positive rights, and democracy. Through reflexive freedom, we can legitimately claim our right to interpret social norms and behaviors, which are not codified juridically and are open to various interpretations of their normative content. However, we can exercise these two different forms of freedom exactly because they can be realized against the backdrop of social freedom, which is a set of social practices and norms that we do not create individually and exist prior to and independent of our individual existence. The norms of cooperation that constitute social freedom are not at our arbitrary disposal because they are embedded in the society we belong to.

Having these considerations in mind, what kind of relevance can Honneth’s viewpoint have for ethical evaluations of the implementation of social distancing and NPIs?

## NPIs as Interpersonal Protection, Care as Recognition

My main argument is that social distancing can be, in specific circumstances, ethically justifiable insofar as it constitutes a coordinated practice that permits the protection of the individual’s health and the population’s health. In this respect, on one hand, it could be argued that care is the realization of a specific form of interpersonal recognition, one diagonal to various social spheres, that constitutes an adequate reaction to the vulnerabilities of our peers. The recognition of others as vulnerable subjects triggers behavior that aims at promoting others’ well-being. On the other hand, it can also be argued that care should be considered a constitutive feature of social freedom. In these circumstances, care through recognition also allows the most vulnerable segments of the population to take part in social activities that would otherwise be dangerous and unhealthy. Therefore, intersubjective care in the form of social distancing should also be seen as a constitutive part of collective protection, which allows even the most disadvantaged to take part in many essential social activities. In light of these considerations, we could say that we have the duty to enact effective forms of social distancing when there is no other way to guarantee participation in public health goods and benefits in a safe way. In a situation in which.

1. Achieving collective herd immunity through natural infections implies harms that are disproportionate, unpriceable, or unpredictable and
2. Individual health cannot be guaranteed by therapies, drugs, or sterilizing vaccines, practices that realize social distancing are ethically justifiable insofar as they produce collective protection grounded in intersubjective forms of care, which mitigate health disparities in societies that are not capable of implementing immediate structural improvements due to resource scarcity.

Considering the important function that role-taking capacity, mutualism, and cooperation play in relation to NPI actuation, Honneth’s paradigm of recognition seems ideal for use in evaluating them. First, such a paradigm can justify social distancing implementation by connecting it not to individual causal responsibility for infection cases. In other words, NPIs would be ethically justifiable insofar as individuals have the duty not to harm others, willingly or not. The temporary restriction of negative and reflective freedom related to social distancing would be justifiable insofar as the collective and simultaneous actions of individuals can work in favor of the achievement of a co-immunity that benefits the worst off. Thus, Honneth’s perspective on intersubjective recognition and social freedom grasps a pivotal point. Given the necessary or contingent impossibility of reaching herd immunity, which could guarantee biological protection to the few individuals that lack natural or acquired immunity, only extensive forms of deep mutualism and social reciprocity can support the achievement of satisfactory collective protection against a pathogen. This scattered mutualism does not imply the simple acknowledgment of NPI measures once they are implemented in a top-down direction. Rather, it requires the active participation of citizens and social groups and adopting a role-taking capacity that allows the justification of NPIs based on the expectations and well-being of others. The basis for the systematic employment of masks does not rely on the idea that masks protect the bearers from the virus. Rather, wearing a mask means that the mask bearer is protecting others from infections she can cause (Eikenberry et al. 2020; Gandhi and Havlir 2020). A similar rationale could be ascribed to other types of NPIs that realize significant forms of social distancing. Citizens should implement them not to protect themselves individually but, rather, to produce, through aggregate actions, outcomes that aim at minimizing the vulnerabilities of their peers of interaction.

It is worth noting that the actuation of such a common agency is different from and more exacting than the simple coordination of self-interested actions. It requires that the agents involved in public policies be willing to act to protect their peers of interaction, despite their private ends and self-interests. This is coherent with Honneth’s idea of recognition. To be realized, good forms of recognition require that recognizers perceive specific normative properties in the recognizees, attribute specific normative and empirical expectations to the same, and satisfy such expectations of the recognizees enacting a behavior that aims at realizing the well-being of the recognizees themselves (Honneth 2007: 329–330). Analogously, a successful realization of NPIs seems to imply that the agents enact social distancing because others have empirical and normative expectations regarding the agents’ conduct. We should enact social distancing insofar as our partners expect that we want to protect them against the risk of infections and they believe, at the same time, that people around them will behave in that manner in situations in which the risk of contagion is high, despite the fact that social distancing can impose limitations on individuals’ freedom and right to self-determination. Coherent with this reading, the moral duty to adopt effective forms of social distancing when necessary is not justified by an external good (saving lives, saving years of life, saving the public health system, an abstract idea of humanity, or the realization of a specific virtue), and it is not anchored to any consequentialist consideration. The actuation of social distancing implies taking the anticipated and foreseeable negative consequences to the person’s life as necessary and sufficient reasons to act. In this respect, caring for somebody is aimed at *promoting* the other’s well-being, becoming almost consistent with utilitarian and consequentialist considerations. Nevertheless, caring as a form of recognition is person centered and does not necessarily entail any calculation concerning the maximization of a specific outcome (lives saved or total years of life gained). Care can thus fluctuate from paternalism, in which adequate care is compatible with bypassing the recipient’s autonomy, to more liberal approaches that aim to assign value to what the recipients of care themselves think is good for them. In both cases, however, the adequate realization of care entails duties that are constitutive of care themselves. Such duties could take the form of either impositions of specific forms of social distancing or the voluntary actuation of NPIs that are sensitive to and limited by the requests of care receivers. In the absence of effective vaccines, forms of social distancing that are enacted in a coordinated way can surely guarantee collective protection against a high level of transmission, a high death rate, and the blockade of healthcare system functionality. Nevertheless, if we adopt the perspective of recognition, the duty to enact NPIs constitutes a good in itself, as determined by the properties we perceived in the care’s receivers but also consistent with the empirical and normative expectations of the same.

Is the realization of recognition as care in contrast with other forms of recognition and social practices that constitute social freedom? At first sight, it seems unlikely that Honneth’s paradigm would attribute any ethical and normative justification to NPIs. These NPIs seem to threaten the realization of those affective and socio-political relations that are indispensable preconditions for achieving individual self-flourishing and psychological well-being. A stay-at-home policy could force many people to drastically diminish the social contacts (relatives, sexual partners, and friends) that constitute their intimate private spheres and carry the burdens of oppressive family relationships without a possibility to escape them. Smart working could be perceived as both an elitist and class privilege by frontline workers and as an unjustified limitation to individual freedom by entrepreneurs and creative and intellectual workers. In both cases, smart working could be perceived as a vector and sign of social disesteem and humiliation. The imposition of such social distancing measures through State coercion could be viewed as an unjustified legal abuse that disrespects civil and human rights, as well as individual rational autonomy and the right to self-determination. Finally, the top-down implementation of NPIs by means of emergency laws and powers seems to betray the idea of democratic self-government that Honneth conceives of as the core of our democratic life and institutions. Nevertheless, things are more complex than this.

### Family and Affective Sphere

According to Honneth, the private sphere of intimate, familiar, and friend relationships is pivotal in the individual achievement of self-confidence. Within this sphere, individuals can freely choose to establish love and care relationships with others only on the basis of their impulses and emotional needs. In this way, they can entertain reciprocal relationships that are based on mutual, disinterested, and empathic attention to the emotions and feelings of the partners of interaction. In this respect, there is no doubt that strong and long-standing implementations of NPIs can weaken and erode our intimate social relationships. However, we should also consider the emotional, psychological, and economic costs of the losses caused by insufficient collective actions against SARS-CoV-2. Between March 2020 and April 2021, more than 1,500,000 children have lost primary caregivers due to COVID-19, including at least one parent or secondary caregiver (grandparents or custodial caregivers). These losses can increase the risk of mental health problems; physical, emotional, and sexual violence; and family economic hardship (Hillis et al. 2021). In this respect, an ethical perspective that considers love and care as fundamental preconditions for individual development cannot disregard the idea that proportionate non-pharmaceutical interventions are preferable to no interventions at all. The loss of parents, relatives, and friends due to COVID-19 represents irrecoverable harm. The weakening and erosion of family ties and friendships, although deplorable, can be healed, although with difficulty.

In addition, the implementation of such measures in the private sphere may be seen as a form of cooperation that leads to the realization of family solidarity and care. I distance myself to protect my beloved, not to harm them. Nevertheless, NPIs that affect private spheres (especially severe lockdown strategies and stay-at-home mandates) should be assessed carefully, considering the specificities of the social contexts in which they are realized. Ghosh et al. (2021) have highlighted how school and non-essential business closures, as well as stay-at-home orders, even if effective in a wider context and for a short amount of time, can contribute to the transmission and spread of the virus among minority groups, which tend to live in crowded multigenerational households due to cultural factors and adverse socio-economic conditions.

Does this consideration imply that not taking any measures or taking only very minor ones can have a more sustainable impact on the same social groups? Aradhya et al. (2021) have provided a study that investigates the causes of excess mortality from COVID-19 among immigrants in Sweden, a country that notoriously preferred to rely on recommendations instead of implementing lockdowns or mask mandates. While “disentangling the role of language barriers and lack of understanding of the healthcare system and recommendations in explaining the excess COVID-19 mortality among immigrants,” Aradhya et al. (2021: 6) found that Swedish people partnered with immigrants experienced higher COVID-19 mortality.

Excess mortality from COVID-19 affects minorities independently of the actuation of lockdown measures or softer policy measures. In fact, coherent with the empirical evidence available, excess mortality seems to be correlated to specific socio-economic factors, which could amplify the incidence of COVID-19 among minorities and disadvantaged groups. In this respect, to argue that policies based only on recommendations and no or soft NPIs are better than lockdown policies, considering the impact they have on the worst off, is inconsistent with the empirical evidence now available.

Another potential objection to the implementation of strong NPIs or lockdowns concerns their impact on the mental health of the population, especially children. Ray et al. (2022) show that, during the first 15 months of the pandemic, youth ER presentations of self-harm, overdose, and hospital admissions decreased by ~ 18% in Ontario. Other data from Ontario (Saunders et al. 2022) show that acute mental health ER and admission levels did not increase during the first 12 months of the pandemic. We have data showing that overall population suicide rates decreased by 32% in Canada in 2020 (McIntyre et al. 2021). Therefore, for now, the idea that the implementation of strict NPIs negatively affects the mental health and well-being of the general population and its youngest cohorts is not scientifically supported.

### Labor Market, Civil Society, and the Economic Sphere

The implementation of NPIs also seems to be justifiable according to the principle of reciprocal esteem that governs civil society and the labor market. Enacting effective social distancing when visiting public spaces can be seen as a form of esteem and respect for frontline workers (nurses, physicians, bus drivers, factory workers, and teachers), who cannot work remotely and are employed in essential services. These workers cannot avoid prolonged exposure to potentially infective contacts and should be allowed to fulfill their professional duties in the healthiest working conditions possible. In this respect, citizens and customers have the shared responsibility to minimize their social contacts as much as they can, while public institutions and private ones (such as corporations) have a collective duty to protect the health of essential workers through the decontamination of workplaces, installing efficient ventilation systems, and providing health insurance that can support workers who have been infected in the workplace. The actuation of NPIs is also normatively relevant for another reason. It protects not only the health of frontline workers but also the good functioning of our productive activities. The uncontrolled spread of infection could cause their partial disruption, harming the process of commodities production and distribution. Thus, non-pharmaceutical interventions constitute an important tool for use in preserving social freedom and the efficiency of social cooperation. Also, the pandemic has highlighted how much workers’ participation and trust are indispensable in realizing efficient public policies. Not only physicians and nurses but also drivers, teachers, cleaners, factory workers, and cashiers are required to expose themselves to a high risk of infection in order to guarantee the basic functioning of social production. Moreover, it is necessary to highlight the fact that the implementation of NPIs is essential in allowing persons who belong to at-risk groups to sustain themselves and continue working safely despite the ongoing pandemic.

Some could argue that, in countries characterized by a widespread informal economy, the actuation of severe NPIs could harm working-class people and low-skilled workers more than COVID-19 illness itself. For instance, a “stay-at-home” order can be implemented more easily by high skilled and non-waged workers than workers with informal jobs. These kinds of occupations often require constant contact with others and are exercised in crowded environments. For this latter category of workers, observing restrictions would mean renouncing the only source of sustenance available to them. It follows that, for them, it would be more advantageous to implement slight NPIs or none at all and that learning to live with the virus would be the best solution for them in terms of costs and benefits. According to this position, the harms produced by unemployment in circumstances in which welfare support is poor or absent would exceed the benefits produced by NPIs in the same conditions. However, this perspective does not consider the following:

1. Infections would impair many workers, driving them to lose their job anyway. In a pandemic such as this one, people can go on working, pretending that everything is fine. However, considering the SARS-CoV-2 transmission rate, its high infectivity, and the number of hospitalizations caused by the virus, the economic consequences would be important and heavy, if not shocking, even if we left the virus free to circulate;
2. Long-COVID, which is associated with SARS-CoV-2 infection, poses risks that are still unquantifiable in the long term. In fact, this is also a situation in which the virus is free to circulate and disrupt socio-economic systems. For instance, long-COVID could worsen labor shortages (Davis et al. 2021).

Nevertheless, especially in the long run, it is undeniable that the socio-economic costs related to NPI implementation can be high and unsustainable for the worst off.

### Sphere of Rights and Democratic Life

To what extent can democratic and liberal states curtail individual freedom and the right to self-determination among their citizens? Can these limitations be seen as an expression of disrespect for the moral autonomy of individuals? Are citizens allowed to participate in the decision-making process regarding preparedness and public health policies through democratic institutions? Surely, NPIs can limit our capacity to fully enact our rights to freedom of movement and assembly. Our interpersonal duty to curb virus transmission can come into conflict with our right to take part in political initiatives and rallies, public meetings, and protests. In addition, the urgent need to enact effective public health measures can alter the democratic discussion in representative democratic systems. Addressing these questions requires considering the difference between urgent support and longer-term crisis preparedness. In periods of non-emergency, governments have time to gather enough scientific evidence before enacting and enforcing laws and policies that could curtail the civil liberties and political rights of their citizens in favor of public health. However, in the last 2 years, we have seen how, once an unexpected situation of emergency arises, even democratic and liberal governments can be forced to make urgent policy maneuvers that impact civil liberties in a vortex of uncertainty: no prolonged deliberation; no legislative debate; and actions taken based on executive orders, pursuant to emergency legislation (Flood et al. 2020: 249–264).

All these aspects, at first sight, seem to be ethically problematic from Honneth’s viewpoint. According to him, in fact, social freedom in the form of democratic participation can be realized by presupposing the following conditions: a juridical system that protects freedom of speech and opinion, a communicative space that allows citizens and social groups interested in political decisions to take part in an informed exchange of opinion, media without conflicts of interest that can inform citizens in a satisfying and clear way, and citizens’ willingness to place the common good before their own private ends (Honneth 2014). In partial contrast to these conditions, in a situation of emergency, democratic institutions may be required to enact the necessary NPIs by bypassing normal democratic mechanisms, balancing between precautionary and proportionality principles, and giving priority to clearness and transparency in the communications with intermediate institutions and citizens. In this regard, it is necessary to specify what proportional measures are when access to public healthcare institutions is severely threatened by the epidemic waves of a potentially disruptive pathogen.

Nevertheless, according to the paradigm of recognition, public authorities could be allowed to ask their citizens to adopt protective behaviors when pandemics, epidemics, and outbreaks pose a threat that the state cannot overcome immediately given its limited resources and incapability to improve social structures immediately. A sudden outbreak of an infectious disease cannot be reasonably handled by public forces, drastically and quickly changing the shape of our public health care systems for many reasons. Hospitals seem to be mainly organized and designed for tackling chronic non-infectious diseases and caring for individual health. Training nurses, anesthesiologists, pneumologists, and the other personnel who are indispensable in caring for patients requires years of higher education. Designing antiviral drugs and vaccines requires time, money, and huge collective efforts as well.

If requesting such a sudden behavioral change is therefore ethically acceptable, is it justifiable to impose social distancing in the form of restrictions that affect both negative freedom and the right to self-determination? In a situation of scarce scientific knowledge about the virological and epidemiological features of the pathogenic agent, epistemic ignorance among citizens, and a widespread lack of practical familiarity with protective behavior, it seems that public authorities can impose restrictions on citizens. However, the imposition cannot last beyond a certain amount of time, which is supposed to be as short as possible. In this respect, especially in the medium and long terms, behavioral changes should be not imposed through coercive tools but, rather, always justified by means of communicative rationality. Public institutions should privilege a communicative approach that explains to citizens what uncertainty is and how to cope with it. Democratic institutions and their representatives should do this without employing fearmongering, using messages hinged on the idea of virtuosity, or blaming citizens for infections they are not morally accountable for. A healthy, authentic democratic country should leave individuals the freedom to collectively enact such a shared moral responsibility, which they should observe as citizens and members of a democratic community. Coherent with this perspective, the ideal manner in which to realize a proper democratic life is dependent on the active and continuous participation of the citizens in the decision-making process, as well as on a constant relationship between citizens, experts, representatives, and institutions. In this regard, there is an important requirement related to this fallibilist approach to democratic practice. Given a specific problem, the agents involved in the decisional process should be ready to accept solutions and alternatives that are efficient and evidence supported, despite their subjective and ideological preferences. This means that NPI imposition could be, in theory, perfectly legitimate from a democratic perspective if there are normative and empirical reasons that can be used to justify it publicly and acknowledged by most citizens (Timmerman 2020; Dahlquist and Kugelberg 2021).

## Limits of the Analysis and Conclusions

What kind of normative considerations can we infer from such an analysis? Honneth’s ideas allow a general ethical justification of NPIs and social distancing as forms of intersubjective protection that sustain social freedom in certain circumstances. Nevertheless, such an ethical approach reminds us that the concrete implementation of these health measures should always be context sensitive. In other words, matters related to health justice should always give priority to the vulnerable and socially disadvantaged when a pandemic is affecting societies characterized by unpreparedness and health injustices. However, the health and normative considerations related to the objective vulnerabilities of these social subjects should always be counterbalanced by the following:

1. Evaluations of social determinants that can make NPIs ineffective or harmful for specific social categories, if not the same social groups NPIs aim at protecting;
2. Evaluations of what the recipients of care think is good for them;
3. Evaluations concerning the physical and psychological distress caused by long-lasting non-pharmaceutical interventions.

These points highlight the fact that the ethical justification of NPIs in every form they can assume is always conditional. Let us consider the extreme case of a stay-at-home policy. Coherent with Honneth’s perspective, a strict lockdown is justifiable insofar as a given society is not prepared or has failed in preventing the spread of a virus among the population. However, the opportunity to implement lockdowns only lasts for a very limited amount of time. They should be eased partially or totally once a constant decrease in infection trajectory is achieved and distress and fatigue indicate that the perceived acceptability of such a measure is decreasing due to mobility reduction, a lack of interpersonal interaction, and psychosocial burden. This is consistent with the most recent scientific discoveries (see Di Domenico et al. 2021a, b).

The previous considerations have several limits. This paper has not attempted to answer questions such as the following: to which extent can NPIs produce social humiliation and marginalization? What are the risks of imposing limitations on public gatherings in terms of disrespect for and violations of civil and political rights? What are the consequences of stay-at-home on the consistency of social relations, individual well-being, and psychological health? Can lockdowns become tools for limiting democratic life and facilitating repression? The implementation of NPIs surely implies many challenges, ethical and political problems, and disadvantages that must be assessed carefully.
